# An Equine-Assisted Intervention Versus Non-Manualized Psychotherapy for Youth in a Residential Childcare Facility

**DOI:** 10.1007/s40653-024-00666-x

**Published:** 2024-10-30

**Authors:** Erika L. Berg, Mike Gooch, Laura M. Feldmann, Bettye Knight, Jess Verlaine, Amber Bach-Gorman

**Affiliations:** 1 Department of Animal Sciences, North Dakota State University, PO Box 6050, Dept 7630, Fargo, ND 58108-6050 USA; 2 Northern Nevada HOPES, Reno, NV USA; 3 Home on the Range, Sentinel Butte, ND USA; 4 Amber Bach-Gorman Counseling and Consulting Services, Fargo, ND USA

**Keywords:** Equine-assisted intervention, Residential childcare, EAGALA, Disruptive behavior disorders, Empathy, Horse

## Abstract

Characteristics of individuals with disruptive behavior disorders often include elevated externalizing behaviors such as impulsivity, defiance of authority and antagonism of social norms. Evidence shows that adolescents diagnosed with these types of disorders are particularly challenging to treat; however, therapies incorporating horses have shown some success. We examined the impact of an equine-assisted intervention compared to a non-manualized cognitive behavioral therapy in youth between 12- and 18-years old living in a residential childcare facility. Upon admittance, youth were administered three assessments: the Youth Self-Report, Adolescent Psychopathology Scale, and Basic Empathy Scale. Youth scoring in the clinical range for at least one externalizing subscale of the Youth Self-Report were assigned to equine-assisted intervention (EQI; *N* = 23) or non-manualized cognitive behavioral therapy (NM-CBT; *N* = 20) groups. Each group received a total of 7 h of treatment per week (three 2-hr group sessions and one 1-hr individual session) for 24 weeks. After 24 weeks of treatment, the same three assessments were administered a second time. There were no significant differences between the NM-CBT and EQI groups in the amount of change between assessments. A significant time effect was found for total externalizing behaviors in the Adolescent Psychopathology Scale and Youth Self-Report such that these measures were within the normal range after 24 weeks of treatment for participants in both groups.

## Introduction

Disruptive behaviors are present in many children and adolescents at some time in their lives and are often manifested in response to stressful situations or events out of their control. However, when those behaviors are exhibited frequently, with extreme intensity or for extended periods of time there is cause for concern. Disruptive, impulse-control and conduct disorders include oppositional defiant disorder (ODD), conduct disorder (CD) and antisocial personality disorder. Disruptive behaviors are often a component of truancy, delinquency, substance use disorders, and interpersonal violence (Deaton, 2008). Additionally, these behavior disorders are characterized by clusters of externalizing behaviors including, but not limited to, aggression, lying, defiance of authority, impulsivity, destructiveness and lack of empathy, with ODD being a common antecedent to CD (American Psychiatric Association, 2013). Furthermore, an estimated 25 to 40% of individuals with CD continue to show behaviors into adulthood that meet the diagnostic criteria for antisocial personality disorder (Murphy et al., 2001). This disorder is characterized by a pattern of violating the rights of others with no regard for their thoughts or feelings, and a marked inability to distinguish between right and wrong (APA, 2013). A large body of evidence suggests that interventions for CD and other severe disruptive behavior disorders during late adolescence and adulthood have limited impact in shifting disruptive behaviors to more pro-social behaviors (Asher & Coie, 1990; Bergman & Magnusson, 1997; Cairns & Cairns, 1994; Caspi et al., 1990; Conner et al., 2006; Dishion at el., 1995; Loeber & Stouthamer-Loeber, 1998; Ollendick et al., 2008; Rohde et al., 1996). The limited efficacy of the applied interventions to behavior change could contribute to negative long-term outcomes in life. Because best outcomes tend to occur with interventions such as Child-Parent Relationship Therapy or Parent Behavior Training prior to age 12, (Kaminski & Claussen, 2017; Weisz & Kazdin, 2010); the development of alternative interventions for older adolescents is crucial.

An etiological construct directly linked to aggression is lack of empathy (Cohen & Strayer, 1996; Miller & Eisenberg, 1988). Empathy is generally defined as sharing another person’s emotional state (Eisenberg & Strayer, 1987). Jolliffe and Farrington (2006) further divide empathy into cognitive and affective components, describing cognitive empathy as the ability to understand the emotions of someone else and affective empathy as the ability to experience the emotions of someone else. It is this ability to empathize with others that make children more receptive to moral teachings (Hoffman, 1975). Numerous studies have confirmed the significant relationships that exist among low levels of empathy, a lack of remorse or emotional response, antisocial behaviors, and aggressive behaviors directed toward humans and animals (Ascione, 1993; Hastings et al., 2000; Jolliffe & Farrington, 2004; Luk et al., 1999). This association is stronger in adolescents and young adults.

Evidence exists to support equine-assisted services as a treatment option for children and adolescents with myriad mental health challenges (Bachi et al., 2011; Lentini & Knox, 2015; Naste et al., 2018; Roberts & Honzel, 2020; Trotter et al., 2008). Equine-assisted services (EAS) is a term that refers to “multiple services in which professionals incorporate horses and other equines to benefit people” (Wood et al., 2021). These services are broadly categorized into therapy, learning, and horsemanship. This study employed both therapy and horsemanship categories (specifically adaptive riding) of equine-assisted services; thus, we describe each of those below as derived from Wood et al. (2021). Therapy incorporating horses can be defined as a professional therapist licensed in their respective field (physical, occupational or speech therapy, counseling or psychotherapy) delivering services within their scope of practice in partnership with horses. Horsemanship is multi-faceted and includes four nontherapy services: (1) adaptive equestrian sports; (2) adaptive riding; (3) driving; and (4) interactive vaulting. Adaptive riding was the horsemanship intervention used in this study. Participants rode horses with adaptations made to tack, equipment or instruction style in order to best support their physical, cognitive, emotional, behavioral or mental health needs. Unmounted or ground-based equine activities (e.g. leading horses through obstacle courses or working with horses ‘at liberty’ with no equipment tethering the horse), as well as grooming and stable management are also included under the umbrella of horsemanship in the context of equine-assisted services management (Professional Association of Therapeutic Horsemanship International, n.d.). It is imperative that every professional offering EAS have the proper skills, credentials and additional training to safely and effectively incorporate equines as an intervention for people of all abilities who are seeking growth and healing with the help of horses.

Psychotherapy incorporating equines is an interactive treatment approach that involves a licensed mental health professional, credentialed equine professional or specialist, and an appropriate equine working together with a client(s) to address treatment goals related to mental health (Professional Association of Therapeutic Horsemanship International, n.d.; Kersten & Thomas, 2004). The Equine Assisted Growth and Learning Association (Equine Assisted Growth and Learning Association (EAGALA) has developed a model of psychotherapy incorporating equines rooted in experiential theory. The EAGALA model is a hands-on, client centered and solution-oriented approach facilitated by a mental health professional and equine specialist, with equine interactions as the cornerstone of the model. An additional tenet of the EAGALA model is that the intervention must be unmounted (no riding), with the horses ‘at liberty’ or untethered by equipment such as a halter or lead rope which allows them to choose whether to engage with clients (Equine Assisted Growth and Learning Association, n.d.). Horses possess many characteristics that make them excellent partners for healing in various settings. As a prey animal, the horses’ ability to read and respond to environmental cues is essential for their survival, and thus they are highly attuned to their surroundings, as well as other creatures near them (Ladewig, 2007; Saslow, 2002). When the horse responds to human behavior during an interaction in a particular space, the behavioral feedback from the horse is immediate. The behavioral reaction of the horse then provides the client with an opportunity to engage in self-reflection, as well as communication with the horse(s) and/or practitioner. These horse-human interactions and subsequent horse-human responses continue throughout a session, providing multiple opportunities for discussion and contemplation.

As herd animals, horses have a social hierarchy within their group and consequently observation of herd dynamics often enables clients to draw parallels with life events and subsequently process those experiences. Furthermore, the horse can act as an emotional buffer between the therapist and client, reducing the confrontation that may be felt in a more traditional one-on-one office session (Ewing et al., 2007). The sheer size and power of the horse also compels one to be present in the moment and may prompt emotions to arise, acting as a catalyst for a therapeutic process (Bachi et al., 2011; Kirby, 2010). In the context of human-equine interactions, Keaveney (2008) has described this phenomenon as “awe,” page 450) or a feeling of great reverence and spirituality associated with the horse. Moreover, Roberts et al. (2004) posits that successfully working with an animal weighing over 1,000 pounds can improve self-confidence. Alternatively, when people come face to face with a creature as powerful as a horse, they may reevaluate their own feelings of power (Irwin, 2001). Finally, the EAGALA model enables clients to use their experience with equines to bring attention to the present and develop self-awareness (Karol, 2007).

A positive association between empathy towards animals and empathy towards humans has been shown (Daly & Morton, 2006; Komorosky & O’Neal, 2015; Poresky, 2015). It has been reported that the stronger the bond between a person and an animal, the greater the degree of empathy exhibited (Poresky & Hendrix, 1990). Evidence also supports the idea that empathy can be learned (Richardson & Norman, 2000). Creating a relationship with a horse in a safe environment may help facilitate both healing (DePrekel & Neznik, 2009) and empathy development (Vidrine et al., 2002), which have been identified as critical characteristics to reduce antisocial behaviors and overt aggression (Bilinsky, 2011; Thompson & Gullone, 2008).

In a meta-analysis of treatment outcomes of cognitive behavioral therapy for anger-related programs in children and adolescents, Sukhodolsky et al. (2004) suggest that treatments teaching actual behaviors were more effective than treatments that target internal constructs thought to be precursors to disruptive behaviors. Although there is strong evidence that cognitive behavioral therapies are effective treatment strategies, there is still room for exploring alternative treatment options that may include elements found in CBT. The experiential nature of equine-assisted services creates a unique therapeutic environment that provides opportunities for feedback and modeling, both behavioral interventions that according to Sukhodolsky et al. (2004), yielded greater effect sizes than their non-behavioral counterparts. Penalva (2021) utilized an interpretive phenomenological analysis to explore the experiences of incarcerated young men participating in psychotherapy incorporating equines. Diagnoses of the incarcerated youth included (but were not limited to) conduct disorder and post-traumatic stress disorder. The following themes were identified by Penalva (2021) from participant interviews and are similar to those that emerged in previously cited works incorporating horses (Chandler, 2005; Kersten & Thomas, 2004; McCormick & McCormick, 1997; Yorke et al., 2008):

1. “Lessons Learned” with subthemes of horsemanship and awareness (pages 72–76).
2. “Traits” with subthemes of confidence and focus (pages 76–78).
3. “Relationship with Others” with a subtheme of communication (pages 78–81).
4. “The Future” with subthemes of life outside incarceration, careers and possibility (pages 81–87).

Additionally, Roberts and Honzel (2020) compared the impact of psychotherapy incorporating equines to traditional group therapy and reported that while both treatment groups improved, youth with serious emotional disturbances who participated in psychotherapy incorporating equines had a significantly greater positive affect both before and after the intervention when compared to youth in a traditional group therapy setting. Finally, work by Stebbins (2012) demonstrated a significant reduction in externalizing behaviors in emotionally disturbed youth who participated in a 10-week horsemanship program.

A limited body of work has investigated the impact of equine-assisted services on youth with disruptive behavior disorders; therefore, the aim of this study was to compare the effects of an equine-assisted intervention (specifically horsemanship and psychotherapy incorporating horses) to a non-manualized cognitive behavioral therapy model for adolescents with elevated externalizing behaviors (e.g. aggression, lying, defiance of authority, impulsivity, destructiveness and lack of empathy) characteristic of individuals diagnosed with disruptive, impulse-control and conduct disorders. We hypothesized that after 24 weeks of treatment, participants receiving the equine-assisted services intervention (EQI) would exhibit a greater reduction in externalizing behavior scores over time compared to those participants in the non-manualized cognitive behavioral therapy (NM-CBT) intervention as reflected by the Youth Self-Report and Adolescent Psychopathology Scales. In addition, we predicted that EQI participants would significantly increase their empathy score compared to NM-CBT as measured by the Basic Empathy Scale.

## Method

### Participants

The participants were adolescents referred to a residential childcare facility in the Northern Plains region of the United States between January 2012 and August 2015. Youth were excluded from admittance to the facility if they had a sex offender conviction; were actively psychotic, suicidal or homicidal; needed detox; or had a Global Assessment of Functioning score less than 40 (the authors recognize that the GAF is no longer used; however, it was during the time of this study). Twenty-four male and nineteen female participants (*n* = 43) between 14 and 17 years old (*M* = 16.1 years, *SD* = 1.0) completed the study. Twenty-eight adolescents identified as white, eight as Native American, four as multiracial, one as African American, one as Hispanic and one as Mexican. All protocols were approved by the North Dakota State University Institutional Review Board #AG11144.

## Procedure

Youth referred to the childcare facility were assessed upon admittance and administered the Youth Self-Report, Adolescent Psychopathology Scale, and Basic Empathy Scale. The inclusion criterion for adolescents in this study was an elevated score for at least one externalizing subscale of the Youth Self-Report (described in the following section). The study had rolling admittance, with participants continually being admitted and discharged throughout the 3 ½ year study duration. Those who met the inclusion criterion were alternately assigned to either an equine (EQI; *n* = 23) or non-manualized cognitive behavioral therapy (NM-CBT; *n* = 20) group. When the EQI group reached its maximum capacity of 6 participants, incoming qualifying residents would be assigned to the NM-CBT group and when a placement opened up in the EQI group, the next resident admitted was placed into the EQI group and so forth. The same three measures (Youth Self-Report, Adolescent Psychopathology Scale and Basic Empathy Scale) were administered a second time after 24 weeks of treatment.

## Measures

### Youth Self-Report

The Youth Self-Report is a 112 item self-report designed to assess behavioral and emotional problems in 11-18-year-old youth (Achenbach & Rescorla, 2001). Estimated time for completion of the Youth Self-Report is 10 min. Using a 3-point Likert scale with 0 = absent, 1 = occurs sometimes, and 2 = occurs often, adolescents rate themselves based on how true each item is currently or within the past 6 months. Syndrome scales were either in the internalizing behaviors domain or the externalizing behaviors domain. Internalizing syndromes include anxious/depressed, withdrawn/depressed, somatic complaints, social problems, and thought problems. Externalizing syndromes include attention problems, rule-breaking behavior, and aggressive behavior. Incoming residents with an elevated T-score of 65 (1.5 standard deviations above normal) or greater on at least one externalizing behavior subscale of the Youth Self-Report were assigned to EQI or NM-CBT groups for the duration of the study. A T-score between 65 and 69 indicates a syndrome in the borderline clinical range, while a T-score of 70 or greater indicates a syndrome in the clinical range.

## Adolescent Psychopathology Scale

The Adolescent Psychopathology Scale is a 346-item, self-report test designed to assess psychopathology, personality problems and social-emotional problems. It has been validated for 12-19-year-old youth and questions are based on DSM-IV symptomatology (Reynolds, 1998). An estimated 45 to 60 min are needed to complete the Adolescent Psychopathology Scale. The Adolescent Psychopathology Scale consists of 40 subscales measuring four domains: Clinical Disorders (20 scales), Personality Disorders (5 scales), Psychosocial Problems (11 scales) and Response Style (4 scales). The number and format of responses varies (e.g. true or false; nearly all the time, sometimes, never or almost never) depending on the questions asked and may include time period as well (e.g. past two weeks, past month, past three months, in general). Examples of questions asked include “I break the rules” and “I feel sorry for myself.” Each subscale has a *T* score which is standardized with a mean of 50 and SD of 10. A higher *T* score = greater psychopathology. A *T* score 1.5 standard deviations above the mean (e.g. *T* score = 65) is considered elevated. The subclinical symptom range is represented by a *T* score of 60 to 64, mild clinical symptoms from 65 to 69, moderate clinical symptoms from 70 to 79 and severe clinical symptoms 80 and above (Reynolds, 1998). Scores from the Clinical Disorders and Personality Disorders domains were used to calculate three broad-based factor score scales measuring Internalizing Disorder, Externalizing Disorder, and Personality Disorder.

### Basic Empathy Scale

The Basic Empathy Scale is a 20-item, self-report instrument that was designed to assess both cognitive and affective empathy and has been validated in adolescents (Jolliffe & Farrington, 2006). Participants answered questions using a 5-point Likert scale from strongly disagree to strongly agree. Examples of questions include “Other people’s feelings don’t bother me at all” and “I can often understand how people are feeling even before they tell me.” The scores can range from 20 (indicating a deficit in empathy) to 100 (indicating a high level of empathy) for total empathy. Time to completion for the Basic Empathy Scale is approximately 10 min.

## Interventions

Both EQI and NM-CBT participants received a total of 7 h of therapeutic intervention each week for 24 weeks, with the intervention type dependent on their assigned treatment group. Sessions were held Monday, Tuesday, Wednesday, and Thursday. To reduce clinician variability, the same two mental health professionals facilitated both interventions (EQI and NM-CBT), rotating days. For example, clinician A would see residents in the EQI group on Monday and Wednesday, while clinician B would see residents in the NM-CBT group on Tuesday and Thursday. The following week clinicians would switch days so clinician A would see residents in the EQI group on Tuesday and Thursday, while clinician B would see residents in the NM-CBT group on Monday and Wednesday, and so forth. The EQI group had two equine specialists as part of the intervention teams as well, one per mental health professional. Any resident with substance use issues also participated in a one hour per week alcohol and drug therapy group, as mandated by state law. In addition, participants in each treatment group ate their meals together three days per week.

## Equine Intervention

The EQI participants engaged in two group psychotherapy sessions and one individual psychotherapy session, all of which utilized equine methods based on the EAGALA model, as well as four hours of horsemanship activities weekly (Table 1). The EAGALA sessions were designed to address psychotherapy goals while the horsemanship activities were created to build confidence through horsemanship skills and enable participants to gain general equine knowledge on proper care, management and feeding of horses. Examples of topics addressed in EAGALA-model psychotherapy sessions included boundaries, problem-solving, overcoming challenges, teamwork, leadership, substance use, empathy, resilience, communication and relationships. As a session example, facilitators would begin each session with a check-in and have various items (e.g. buckets, pool noodles, ribbons, balls, pylons, ropes) available in the arena space. They would then invite clients to build a path representing obstacles in their life and then move a horse, or group of horses, through the path of their life. After the interaction with the horse(s), the client processes the experience with the facilitators.

**Table 1:** Weekly schedule for the EQI group

	Monday	Tuesday	Wednesday	Thursday
**1st Hour**	Group Horsemanship Activities	Group Horsemanship Activities	Individual EAGALA sessions	Group Horsemanship Activities
**2nd Hour**	Group meal	Group meal		Group meal
**3rd Hour**	Group EAGALA	Group EAGALA		Group Horsemanship Activities

Horsemanship activities followed a knowledge-based curriculum designed by the first three authors and included both mounted and unmounted work. Participants learned about equine nutrition and anatomy, saddle and bridle parts, and horse behavior and health care. Unmounted work included how to properly catch and handle horses, grooming and brushing techniques, leading horses with a halter and rope, and working horses around obstacles from the ground. Mounted work consisted of learning correct and effective riding position, understanding cues for different gaits and maneuvers, and practicing moving horses through patterns. Discussions on the importance of maintaining a calm emotional state when working with horses, coupled with breathing techniques to aid in self-regulation of emotions were practiced as well.

### Non-manualized Cognitive Behavior Therapy Intervention

Cognitive behavioral therapy (CBT) is an approach used to assist the client in identifying the link between negative or distorted cognitions often associated with external stimuli and the subsequent internal emotion and resulting behavioral response (Rice, 2015). The goal is to identify the distorted or illogical thought (cognition) and replace it with more positive self-statements or less distorted interpretations of the stimuli. A good example in the milieu might be something to the effect of “a girl said something mean to me so I must be a bad person.” This results in an emotional response such as feeling sad or mad and behaving in a self-destructive way (e.g. angry outbursts). A CBT approach would help the client recognize and reframe the distorted thought to something like “she may have said something mean to me, but that does not imply I am bad. Maybe she is just having a bad day or it just came out wrong”.

Manualized CBT involves the implementation of a structured treatment program that includes a predetermined set of components (e.g. psychoeducation, cognitive restructuring, graded exposure) delivered to clients in a temporal pattern within a certain number of sessions that includes homework outside of the session (Hoyer et al., 2017). Non-manualized CBT is a less structured approach that allows more flexibility in the delivery of the intervention. Clinicians at the residential childcare facility in this study previously attempted to incorporate manualized interventions; however, it was often difficult to maintain the rigid structure required of manualized delivery when working with highly emotionally reactive youth who were seemingly living in a state of constant stress. Consequently, a non-manualized CBT intervention was developed.

Each week, the NM-CBT participants received non-manualized cognitive behavioral therapy for six hours of group sessions and a one-hour individual session (Table 2). Session topics varied by week and were similar to those addressed in the EAGALA-model psychotherapy sessions in the EQI group. Activities were implemented to identify distortions in thought processes, as well as alternative, healthy coping strategies to mitigate distress. Sessions included additional motivational techniques, similar to ones in the EQI group, that were designed to empower the participants to utilize self-monitoring, breathing and self-soothing to improve their ability to regulate emotions.

**Table 2:** Weekly schedule for the NM-CBT group

	Monday	Tuesday	Wednesday	Thursday
**1st Hour**	Group therapy	Group therapy	Individual sessions	Group therapy
**2nd Hour**	Group meal	Group meal		Group meal
**3rd Hour**	Group therapy	Group therapy		Group therapy

## Results

A repeated measures analysis of variance (ANOVA) was used to analyze the data, using the mixed procedures of SAS (version 9.4; SAS Institute Inc.). The model included the effects of gender, time, treatment, and time × treatment interaction. Least square means were generated for each effect. There were no significant effects (*p* > 0.05) of gender for any of the measures. A significant effect of time was found on the Externalizing Disorder Factor score of the Adolescent Psychopathology Scale (*F* [1,41] = 14.25, *p* < 0.01) such that the mean week 24 score (55.03, *SE* = 1.75) was significantly lower than the mean week 0 score (63.78, *SE* = 1.75). Similarly, the Total Externalizing Score of the Youth Self-Report (*F* [1,41] = 26.68, *p* < 0.01) showed a significant time effect such that the mean week 24 score (62.18, *SE* = 1.24) was significantly lower than the mean week 0 score (70.18, *SE* = 1.24) as well. These results indicate a significant reduction in externalizing behaviors for participants in both EQI and NM-CBT groups over 24 weeks of treatment. No differences were seen over time or between treatments for the Internalizing Disorder Factor scores (*F* [1,41] = 0.58, *p* = 0.45; *F* [1,39] = 0.07, *p* = 0.80) or the Personality Disorder Factor scores (*F* [1,41] = 0.13, *p* = 0.72; *F* [1,39] = 0.20, *p* = 0.66) of the Adolescent Psychopathology Scale, respectively. Similarly, no differences were seen over time or between treatments for the Total Internalizing Score of the Youth Self-Report (*F* [1,41] = 2.68, *p* = 0.11; *F* [1,39] = 0.07, *p* = 0.79). A significant difference was found for the cognitive scale of the Basic Empathy Scale (*F* [1, 39] = 4.80, *p* = 0.03) such that the NM-CBT group had a greater mean score (37.25, SE = 0.99) than the EQI group (34.29, *SE* = 0.89). However, no differences were seen over time (*F*[1,41] = 1.95, *p* = 0.17) for the cognitive scale, nor were there any differences seen over time (*F*[1,41] = 1.56, *p* = 0.22; *F*[1,41] = 2.43, *p* = 0.13) or between treatments (*F*[1,39] = 0.17, *p* = 0.69; *F*[1,39] = 0.48, *p* = 0.49), for the affective scale or total score of the Basic Empathy Scale, respectively. There were no significant time x treatment interactions (*p* > 0.05) for any of the measures in this study (Adolescent Psychopathology Scale, youth Self-Report or Basic Empathy Scale).

## Discussion

The objective of this study was to determine the impact of an equine intervention compared to non-manualized cognitive behavioral therapy over 24 weeks for youth with elevated externalizing behaviors living in a residential treatment facility. Specifically, we hypothesized that after 24 weeks of treatment, participants receiving the EQI would demonstrate a greater reduction in externalizing behavior scores over time compared to those participants in the NM-CBT intervention. Contrary to our hypothesis, we did not find a difference between the outcomes for youth in the equine intervention compared to the non-manualized cognitive behavioral therapy group; however we did see a reduction in externalizing behaviors of youth in a residential childcare facility in both groups. Mueller and McCollough (2017) who investigated the impact of 10 weeks of equine-facilitated psychotherapy compared to existing traditional therapeutic services for youth ages 10–18 years old who had experienced trauma, found a reduction of PTSD symptomology in both groups. While PTSD symptomology or diagnosis was not included in our study, all youth in the current study had some degree of trauma history which included verbal, physical, and/or sexual abuse. Psychotherapy, and specifically cognitive behavioral therapy (CBT), is recognized as a treatment option for individuals with externalizing disorders such as ODD and CD (Battagliese et al., 2015; Lochman et al., 2011; Sukhodolsky et al., 2004), children with trauma and behavior problems (Cohen et al., 2010), as well as for treating adults with PTSD (Lonergan, 2014; Watkins et al., 2018). It is noteworthy that in our study and the Mueller and McCollough (2017) study a reduction in externalizing behaviors and PTSD symptomology was seen, respectively, with both studies incorporating an equine intervention and traditional therapeutic services. While CBT is regarded as a primary treatment option, it is not an effective intervention for all clients (Bryant et al., 2010; Enoch, 2013) and can produce negative affect in some instances (Brown et al., 2018). Consequently, alternative strategies that show promise, such as equine interventions, should be investigated further.

The authors acknowledge multiple alternative explanations for the significant reduction in externalizing behaviors for participants in both EQI and NM-CBT groups in this study. One explanation is a statistical phenomenon called regression to the mean. Regression to the mean postulates that individuals identified as being “high risk” (e.g. youth entering a residential facility when their externalizing behaviors are greatly escalated, p. 1) will tend to improve at a later time point due to the laws of probability (Linden, 2013). When an intervention is introduced and improvements are observed, it can lead to the erroneous conclusion that these changes are due to the treatment when in fact improvements are likely even without an intervention (Bland & Altman, 1994). A second explanation is that the youths’ behavior improved through positive, supported choices they made and not as a result of the intervention. Finally, the reduction in externalizing behaviors observed could be the result of participants being placed in a stable and more supportive environment than their home or the foster care system. Additionally, the residential setting allows for greater monitoring of youth behaviors and significantly reduces the likelihood of individuals engaging in risky activities.

For youth with disruptive behavior disorders, treatment is challenging and interventions often have limited long-term success (Conner et al., 2006; Ollendick et al., 2008). In a 2017 review article, Kaminski and Claussen concluded that group parent behavior therapy as well as individual parent behavior therapy with child participation met the criteria to be considered “well-established treatments” for children with disruptive behavior disorders; however, this work focuses on children 12 and younger and does not include treatment recommendations for youth living in foster care or residential facilities. One therapeutic model developed for a comparative age group is Multisystemic Therapy. Multisystemic Therapy is an evidence-based treatment developed specifically for ages 12–17 years with serious antisocial behavior concerns. The model addresses multiple systems that influence youth and families such as, but not limited to, their family systems, neighborhoods, and schools (Henggeler et al., 2009; McCart & Sheidow, 2016).

Treatment Foster Care Oregon is also a well-established treatment for adolescents age 12 to 19-years with disruptive behavior disorders (Chamberlain, 2003; McCart & Sheidow, 2016). This program focuses on five main treatment areas: increasing prosocial behaviors while decreasing negative behaviors and supporting engagement in educational settings, peer group monitoring, creating clear expectations and daily structure, increasing positive relationship skill building, and increasing parenting skills with the intention to decrease family conflict. However, both models are home or family-based interventions and do not directly address the unique needs of youth in residential facilities. It has been reported that multimodal interventions have found some success with this population (Battagliese et al., 2015; Taiwo & Osinowo 2011); thus, incorporating equine work into a treatment strategy may be beneficial.

Incorporating equine interventions into psychotherapy treatment plans for individuals who have not responded to more traditional office-based therapies has been documented. In a review article, Lentini and Knox (2015) indicated a common theme for incorporation of equine into psychotherapy treatment was the disengaged and resistant client, as well as those abused or neglected youth who did not respond to traditional one-on-one office-based therapy. Horses have also been shown to be a motivating factor for attendance to group psychotherapy sessions (Berg et al., 2021). Trotter et al. (2008) found at-risk children and adolescents to respond significantly better in multiple measures to an equine-assisted counseling intervention compared to an in-class, school-based counseling program. Incorporating equines into a treatment session provides an experiential opportunity for clients to gain self-awareness (McCormick & McCormick, 1997) and confidence (Chandler, 2005; Kersten & Thomas, 2004), experience trust (Bachi et al., 2011; Vidrine et al., 2002), work on emotional congruence (Perkins, 2018; Stiltner, 2013), and recognize how their physical movement and behaviors can impact others based on how they interact with the equines present, and in turn how those equines choose to interact with them. The impact of the human-equine bond and moments of self-realization from interactions with non-judgmental equines are often quite significant from the client’s perspective (Vidrine et al., 2002; Yorke et al., 2008).

Anecdotally, practitioners in the current study frequently noted that individual challenges rise to the surface more quickly for clients engaged in an equine session compared to those engaged in office-based therapy without the presence of horses. One benefit to this may be that clients are able to address those challenges sooner and move forward more quickly. For future 24-week studies we would recommend a mid-point assessment (12 weeks) to capture whether differences existed in how quickly externalizing behaviors were normalized in each group. Certainly, finding alternative and efficacious solutions for youth with disruptive behavior disorders living in residential facilities is paramount to support them as they transition into adulthood and society at large.

We predicted that participants in the EQI group would have greater empathy scores over time compared with the NM-CBT group; however, this was not the case. Neither the EQI or NM-CBT demonstrated a significant increase in cognitive, affective or total empathy over time; however, the NM-CBT group did have a greater overall cognitive empathy score. This may have been influenced by the EQ group’s lower initial cognitive empathy scores as compared to NM-CBT. In other words, the NM-CBT group started with higher cognitive empathy scores that neither declined nor improved over time. The lack of improved empathy in the EQI group was unexpected as working with horses has been shown to increase empathy in youth at-risk (Deaton, 2008; Ho et al., 2017), as well as typically developing adolescents (Pelyva et al., 2020). Similar to our results however, Ewing et al. (2007) found no difference in empathy of youth with severe emotional disorders who participated in an equine facilitated learning program. Additionally, we saw no difference between genders, which was surprising, as females frequently are found to have significantly greater empathy than males (Jolliffee & Farrington, 2006; Salas-Wright et al., 2013; Zych et al., 2022).

An oft-cited gap in this field of study is the lack of rigor in study design (Kruger & Serpell, 2010; Kruger et al., 2004), as well as a lack of manualized treatment strategies specific to incorporating equines into a therapeutic intervention (Arnon et al., 2020); however, work in this area is ongoing. Gergley (2020) developed an EAGALA Treatment Integrity Measure (ETIM) to evaluate the fidelity of the EAGALA model for research and clinical purposes, helping to ensure consistency of the model’s use by practitioners, in addition to allowing comparison between studies. Arnon et al. (2020) recently published preliminary data for a standardized treatment protocol for group equine-assisted therapy treatment for PTSD (EAT-PTSD) for the veteran population. Naste et al. (2018) described an equine intervention for treatment of youth with complex trauma called Equine Facilitated Therapy for Complex Trauma which incorporates an evidence-based framework with Attachment, Regulation and Competency. Johansen et al. (2014) developed a manualized equine-facilitated body and emotion-orientation psychotherapy for those individuals who have not found success with conventional psychotherapy interventions. Finally, Acri et al. (2021) developed Reining in Anxiety, a manualized protocol for the adaptive/therapeutic riding setting that incorporates cognitive behavioral elements specific to addressing mild to moderate anxiety in youth. Manualizing protocols gives researchers the opportunity to more directly compare studies and raises the integrity of the intervention.

### Limitations

In this study we did not find a difference between the outcomes for youth in the EQI compared to the NM-CBT group; however, we did see a reduction in externalizing behaviors of youth in a residential childcare facility in both groups. We acknowledged alternative explanations for these differences, as well as other limitations to our study. Confounding factors included incorporation of two equine interventions simultaneously (horsemanship and psychotherapy incorporating equines); changing group dynamics due to staggered, rolling enrollment; and the presence of two practitioners for the EQI group but only one for the NM-CBT group (albeit the same practitioners). Incorporating two equine interventions in the same treatment group did not allow us to determine whether one equine intervention was superior to another for this population or whether simply time with the horses was the greatest influence. Furthermore, while the instruments we used to measure change were validated for the population studied, all were self-report measures and therefore the potential for bias in reporting exists (Althubaiti, 2016). Finally, because the research was performed at a working, residential ranch with rolling enrollment whose priority was the well-being of the youth under their care, randomization of treatment groups was not possible.

## Conclusion

The authors acknowledge that more research is needed to investigate the efficacy of equine interventions for this population of youth. Incorporating a qualitative component exploring the role equines may play in relation to empathy and reduction of disruptive behaviors is warranted. The results of a qualitative inquiry may provide insights into future constructs to be investigated, in addition to instrument and assessment tools that may best assess aforementioned constructs. Second, a solid theoretical framework of incorporating equines into treatment plans for youth with elevated externalizing behaviors would create clarity in connecting specific interventions (e.g. horsemanship vs. psychotherapy incorporating equines) to specific behavior changes and would increase study fidelity. Additionally, the specificity of a model would ensure consistent application of the intervention across practitioners and environments, increasing study reliability. Lastly, determining optimal data collection points and assessment frequency may help to elucidate rate of change within treatment progression.

## Data Availability

The participants in this study did not give consent for their data to be shared publicly, therefore the data supporting this research is not available.
